# Synthesis and Evaluation of Novel Norfloxacin Isonitrile ^99m^Tc Complexes as Potential Bacterial Infection Imaging Agents

**DOI:** 10.3390/pharmaceutics13040518

**Published:** 2021-04-09

**Authors:** Si’an Fang, Yuhao Jiang, Di Xiao, Xuran Zhang, Qianqian Gan, Qing Ruan, Junbo Zhang

**Affiliations:** Key Laboratory of Radiopharmaceuticals, Ministry of Education, College of Chemistry, Beijing Normal University, Beijing 100875, China; 201531150045@mail.bnu.edu.cn (S.F.); 201921150103@mail.bnu.edu.cn (Y.J.); 202031150054@mail.bnu.edu.cn (D.X.); zhangxr@bnu.edu.cn (X.Z.); 201731150054@mail.bnu.edu.cn (Q.G.); 201931150055@mail.bnu.edu.cn (Q.R.)

**Keywords:** norfloxacin, isonitriles, quinolones, technetium-99m, infection imaging

## Abstract

To develop potential technetium-99m single-photon emission computed tomography (SPECT) imaging agents for bacterial infection imaging, the novel norfloxacin isonitrile derivatives CN4NF and CN5NF were synthesized and radiolabeled with a [^99m^Tc][Tc(I)]^+^ core to obtain [^99m^Tc]Tc-CN4NF and [^99m^Tc]Tc-CN5NF. These compounds were produced in high radiolabeling yields and showed hydrophilicity and good stability in vitro. The bacterial binding assay indicated that [^99m^Tc]Tc-CN4NF and [^99m^Tc]Tc-CN5NF were specific to bacteria. Compared with [^99m^Tc]Tc-CN4NF, biodistribution studies of [^99m^Tc]Tc-CN5NF showed a higher uptake in bacteria-infected tissues than in turpentine-induced abscesses, indicating that [^99m^Tc]Tc-CN5NF could distinguish bacterial infection from sterile inflammation. In addition, [^99m^Tc]Tc-CN5NF had higher abscess/blood and abscess/muscle ratios. SPECT image of [^99m^Tc]Tc-CN5NF showed that there was a clear accumulation in the infection site, suggesting that it could be a potential bacterial infection imaging radiotracer.

## 1. Introduction

In the 18th and 19th centuries, public health has developed significantly. By the 20th century, the treatment of infections had been greatly improved due to the discovery of antibiotics. However, currently, in many countries, the overuse of antibiotics has led to the appearance of drug-resistant bacteria, which greatly restrict the effect of the use of antibiotics. Bacterial infections have become one of the highest causes of morbidity and mortality in the world, especially in developing countries [1]. Moreover, it is predicted that by 2050, antibiotic-resistant infections will become the leading cause of death in humans [2]. Taking into account that patients with inflammation may develop different diseases at the same time, clinical testing may not always detect a clear cause of the inflammation, which will lead to delays in treatment and the abuse of antibiotics. Therefore, the early diagnosis of infection and judgment of the types of infections can bring timely, precise, and appropriate treatment to patients, which can minimize the excessive use of antibiotics for patients [3,4].

Clinically, structural imaging techniques, such as magnetic resonance imaging (MRI) and computed tomography (CT), can be applied in the diagnosis of infection. However, these imaging tools rely on anatomical changes in the late course of the disease. Therefore, in the early stage, when morphological changes cannot be clearly observed, infected lesions cannot be effectively detected [5]. Compared with CT and MRI, nuclear medicine technologies, such as positron emission tomography (PET) and single-photon emission computed tomography (SPECT), are based on metabolic changes due to physical, chemical, and biochemical alterations in organs. These technologies can detect lesions at an early stage [6,7,8]. Radiopharmaceuticals with high specificity can be selectively concentrated to the site of infection, leading to the accurate detection of pathogens and the rapid, appropriate treatment of patients. At present, the distinction between infection and sterile inflammation is of great significance. A variety of radiopharmaceuticals have been developed to detect inflammation [9,10,11,12,13,14,15,16,17,18,19]. For example, ^18^F-2-Fluoro-2-deoxy-D-glucose ([^18^F]FDG) have already been used for infection imaging in clinics. However, it cannot distinguish between bacterial infection and sterile inflammation [10]. Radiolabeled 1-(2′-deoxy-2′-fluoro-β-D-arabinofuranosyl)-5-iodouracil (FIAU) ([^124^I]-FIAU) PET imaging cannot sensitively and specifically detect bacterial infection in pulmonary or musculoskeletal systems [16]. There is still a need to develop new radiopharmaceuticals for bacterial infection imaging in nuclear medicine.

Technetium-99m is widely used in nuclear medicine due to its low cost, physical properties, including half-life (6 h) and photon energy (140 keV), and ability to bind to biologically active molecules through bifunctional chelating agents. To date, there have been several antibacterial agents labeled with ^99m^Tc [20,21,22,23]. Among them, [^99m^Tc]Tc-ciprofloxacin has been widely evaluated by academic and medical groups worldwide. [^99m^Tc]Tc-ciprofloxacin has good infection specificity, a low preparation cost, and good imaging quality, and it can be prepared by kits. However, [^99m^Tc]Tc-ciprofloxacin has some limitations, such as a low radiochemical yield, and the final product requires additional purification [24]. In the structure of ciprofloxacin, the carbonyl and carboxyl groups are considered to be essential pharmacophores that may coordinate with technetium-99m, so labeling with ^99m^Tc will reduce its binding affinity to bacteria [25].

Norfloxacin is an important antibacterial agent in clinical applications and shows significant antibacterial effects on both Gram-positive and Gram-negative bacteria. In 2011, the [^99m^Tc]TcN norfloxacin dithiocarbamate complex was successfully prepared as a targeted agent for infection imaging [26]. It exhibits potential as a bacteria-specific infection imaging agent. However, significant radioactivity accumulation was found in the liver (28.52 ± 1.32% ID/g at 4 h post-injection) and lungs (22.87 ± 5.82% ID/g at 4 h post-injection).

Isonitrile (CN-R) is a bifunctional monodentate ligand that can coordinate with a [^99m^Tc]Tc(I) core to form stable octahedral complexes in high yield, such as [^99m^Tc][Tc(CN-R)_6_]^+^ [27]. The ^99m^Tc complex containing six isonitrile ligands can be successfully obtained with great stability and inertness. In the study of novel bifunctional radiopharmaceuticals, the linker moiety between the chelator and the targeting vector usually affects the pharmacokinetic properties of the complexes. For example, hydrocarbon chains (CH_2_)_n_ as linker moieties can change the lipophilicity of the complex, which leads to diverse pharmacokinetics and biodistribution of the radiotracers.

**Figure 1:**
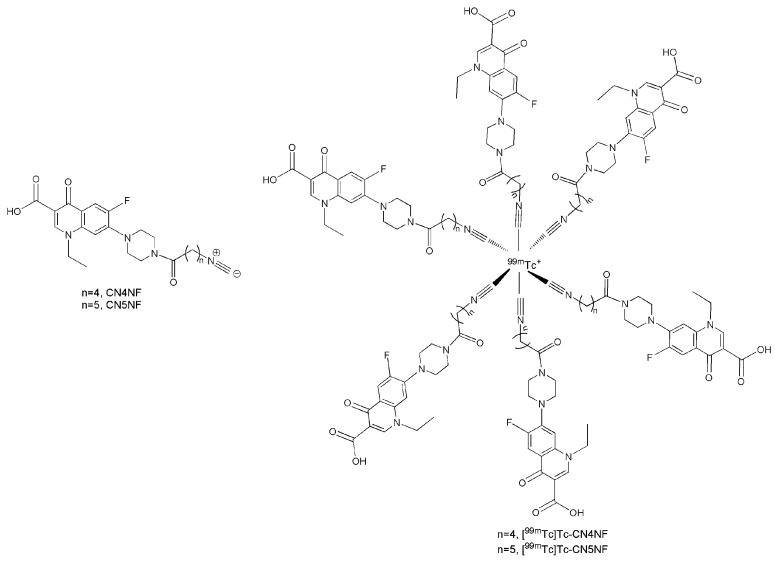
Structures of CNnNF (*n* = 4 or 5) and [^99m^Tc]Tc-CNnNF (*n* = 4 or 5).

The piperazinyl group of norfloxacin can react with different isonitrile-containing active esters to obtain different isonitrile derivatives. According to the background discussed above, in this study, to develop novel ^99m^Tc-labeled radiotracers for bacterial infection imaging, two norfloxacin derivatives with isonitrile groups (CN4NF and CN5NF) were synthesized and labeled with ^99m^Tc to prepare [^99m^Tc]Tc-CN4NF and [^99m^Tc]Tc-CN5NF (Figure 1). The possibilities of these two radiopharmaceuticals as potential bacterial infection imaging agents were evaluated.

## 2. Materials and Methods

### 2.1. Materials

Norfloxacin was purchased from J&K chemical, Beijing, China. All other reagents were of reagent grade and were used without further purification. [^99m^Tc]NaTcO_4_ was obtained from a ^99^Mo/^99m^Tc generator purchased from Atomic High Tech Co. Ltd., Beijing, China. NMR spectra were recorded on a 400 MHz and 600 MHz JNM-ECS spectrophotometer (JEOL, Tokyo, Japan). ESI-MS spectra were recorded on an LC–MS Shimadzu 2010 series. HPLC analysis was performed on a Waters 2489 UV system and a Gabi raytest radioactivity detector using an analytical column (C-18, Kromasil, 100–5 μm, 250 × 4.6 mm). The centrifuge model was 800-1 (Ronghua, Jintan, China). The radioactivity was measured by WIZARD2 2480 Automatic Gamma Counter (PerkinElmer, Waltham, MA, USA), an HRS-2000 technetium analyzer (Huaruisen, Beijing, China), and RW-905A radioactivity meter (Hechang, Beijing, China). SPECT/CT imaging was performed on micro SPECT/CT equipment with 930 collimator (Trifoil, Chatswort, CA, USA). The SPECT technical parameters were as follows: native resolution ≤ 1.6 mm; detection area ≥ 128 mm × 128 mm; and sensitivity ≥ 4800 cps/MBq. Animal studies were performed in accordance with the Regulations on Laboratory Animals of Beijing Municipality and the guidelines of the Ethics Committee of Beijing Normal University (permit no. CLS-EAW-2018-001, June 2018).

### 2.2. Synthesis of Norfloxacin Isonitriles (CN4NF and CN5NF)

**Scheme 1:**
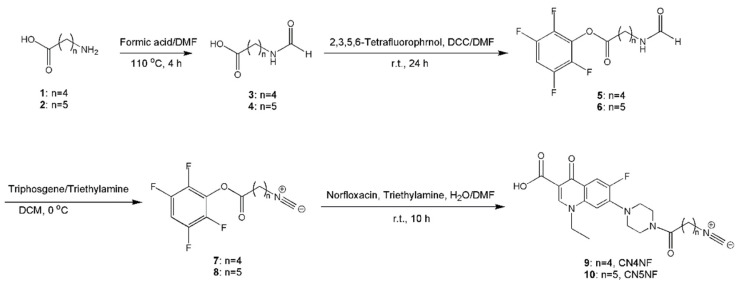
Synthetic route of CN4NF and CN5NF.

Compounds **7**–**8** were synthesized according to the reported literature [28], and the reaction route is shown in Scheme 1. The general procedure for the preparation of **9**–**10** was as follows. Norfloxacin (160 mg, 0.5 mmol) and triethylamine (101 mg, 1 mmol) were dissolved in 5 mL of DMF and 5 mL of water and then stirred at room temperature for 30 min. Compounds **7**–**8** (0.5 mmol) were added to this stirred solution, and the reaction mixture continued to stir at room temperature for 10 h. The reaction mixture was extracted from the aqueous phase with CH_2_Cl_2_ (50 mL × 3). The organic layer was washed with water (50 mL × 3), dried over sodium sulfate and evaporated under reduced pressure at 50 °C. Then, silica gel column chromatography (5% MeOH/CH_2_Cl_2_), full purification, and drying were used to obtain compounds **9**–**10** as white powders.

Compound **9** (CN4NF, 103 mg, yield 48.13%). ^1^H-NMR (600 MHz, CDCl_3_) δ (ppm): 14.93 (s, 1H), 8.67 (s, 1H), 8.09 (d, J = 12.7 Hz, 1H), 6.84 (d, J = 6.7 Hz, 1H), 4.31 (q, J = 7.3 Hz, 2H), 3.86 (s, 2H), 3.71 (s, 2H), 3.45 (dd, J = 8.6, 3.8 Hz, 2H), 3.34 (s, 2H), 3.27 (s, 2H), 2.45 (t, J = 7.0 Hz, 2H), 1.85 (dt, J = 14.0, 6.9 Hz, 2H), 1.57 (dd, J = 15.1, 7.7 Hz, 5H). ESI-HRMS for C_22_H_26_FN_4_O_4_ [M + H]^+^: calcd. 429.1938, found 429.1932.

Compound **10** (CN5NF, 89 mg, yield 40.27%). ^1^H-NMR (400 MHz, CDCl_3_) δ (ppm): 8.63 (s, 1H), 8.03 (d, J = 12.8 Hz, 1H), 6.83 (d, J = 6.8 Hz, 1H), 4.31 (q, J = 7.2 Hz, 2H), 3.91–3.79 (m, 2H), 3.70 (d, J = 4.8 Hz, 2H), 3.48–3.37 (m, 2H), 3.34 (d, J = 4.6 Hz, 2H), 3.30–3.23 (m, 2H), 2.41 (t, J = 7.3 Hz, 2H), 1.71 (dt, J = 15.0, 7.4 Hz, 5H), 1.57 (t, J = 7.2 Hz, 4H). ESI-HRMS for C_23_H_28_FN_4_O_4_ [M + H]^+^: calcd. 443.2095, found 443.2089.

### 2.3. Radiolabeling

In a 10 mL penicillin vial, 1 mg of sodium citrate and 50 µL of SnCl_2_·2H_2_O (1 mg/mL) were mixed, and the pH of the solution was adjusted to 8 with NaOH (0.1 M). Then, 100 μL of norfloxacin isonitrile solution (1.0 mg of ligand per 1.0 mL of DMSO solution) was added to the solution. Next, freshly eluted [^99m^Tc]NaTcO_4_ solution (37–74 MBq) was added to the vial. The reaction mixture was heated at 100 °C for 30 min and cooled to room temperature.

The radiochemical purities of [^99m^Tc]Tc-CN4NF and [^99m^Tc]Tc-CN5NF were determined by thin layer chromatography (TLC) and high-performance liquid chromatography (HPLC). TLC was carried out on glass microfiber chromatography paper impregnated with silica gel (iTLC-SG) and eluted with anticoagulant acid citrate dextrose solution (ACD solution). HPLC analysis was performed with an analytical column at a flow rate of 1.0 mL/min (solvent A: purified water with 0.1% trifluoroacetate, solvent B: acetonitrile with 0.1% trifluoroacetate; 0–2 min, 10% B; 2–10 min, 10–90% B; 10–18 min, 90% B; 18–25 min, 90–10% B).

### 2.4. Stability Study

The stabilities of [^99m^Tc]Tc-CN4NF and [^99m^Tc]Tc-CN5NF were evaluated by measuring the radiochemical purity (RCP) of each complex. The RCP was determined by TLC of the mixture after remaining in saline at room temperature for 6 h and after treatment in mouse serum at 37 °C for 6 h.

### 2.5. Partition Coefficient Measurement

In order to evaluate the hydrophilicity of the complexes, 0.1 mL of the radiolabeled complex was mixed with 0.9 mL of phosphate buffer (0.025 mol/L, pH 7.4) and 1 mL of 1-octanol in a centrifuge tube. The mixture was shaken on a vortex mixer at room temperature for 1 min and centrifuged at 5000 r/min for 5 min afterwards. Aliquots of 100 µL of both 1-octanol and PBS were collected and the radioactivity was determined with a gamma counter. The partition coefficient was calculated as follows: *p* = (counts per minute in 1-octanol/counts per minute in phosphate buffer). Each group of measurements are carried out three times in parallel. The log *p* values were expressed as the mean ± SD.

### 2.6. In Vitro Bacterial Binding Study

The in vitro bacterial binding of [^99m^Tc]Tc-CN4NF and [^99m^Tc]Tc-CN5NF was evaluated by using a previously reported method [29]. In general, 0.1 mL of 3.7 MBq radiolabeled complex solution and 0.1 mL of PBS (pH 7.4) containing approximately 1 × 10^8^ *Staphylococcus aureus* were added to a test tube with 0.8 mL of saline. The mixture was incubated for 60 min at 37 °C, and then the tubes were centrifuged at 8000 r/min for 5 min. Afterwards, the pellets were washed with PBS (pH 7.4, 1 mL × 3). The removed supernatant and bacterial pellets were collected, and the radioactivity was measured with a gamma counter. The background tube was an incubation with no bacteria added. Each group of measurements were carried out six times in parallel. The bacteria binding value was calculated as follows:

Bacteria binding% = (cpm in precipitate − cpm in background)/(cpm in precipitate − cpm in background + cpm in supernatant) × 100%.(1)

To determine the specificity of [^99m^Tc]Tc-CN4NF and [^99m^Tc]Tc-CN5NF binding to bacteria, norfloxacin was used as an inhibitor. The bacteria were preincubated with 0.1 mL of norfloxacin (1 mg/mL) for 1 h at 37 °C. Then, the radiolabeled complexes were added followed by incubation for 1 h at 37 °C. The bacteria binding value was calculated as described above. Each group of measurements are carried out six times in parallel. The results are expressed as the mean ± SD.

### 2.7. Biodistribution Study in Bacteria-Infected Mice

First, 0.1 mL of a 1 × 10^8^/mL suspension of *S. aureus* was injected into the left thigh muscle of female Kunming mice (18–22 g). Animal studies were performed according to the principles of laboratory animal care and the guidelines of the Ethics Committee of Beijing Normal University (permit no. CLS-EAW-2018-001, June 2018). Approximately 3–5 days later, the mice were injected with 7.4 × 10^5^ Bq of the ^99m^Tc-labeled complexes ([^99m^Tc]Tc-CN4NF or [^99m^Tc]Tc-CN5NF) via the tail vein. The mice were sacrificed at 0.5, 2, and 4 h postinjection. The organs of interest (the infected section, normal muscle in the right thigh, blood, heart, liver, lung, kidney, spleen, stomach, bone, and small intestine) were collected, weighed, and quantified for radioactivity. The results are expressed as the percentage of injected dose per gram of tissue (% ID/g). After the mice were sacrificed, the tissues containing thyroid around the trachea in the neck were taken and quantified for radioactivity. The results are expressed as the percentage of injected dose (%ID).

### 2.8. Biodistribution Study in Turpentine-Induced Abscess Mice

Turpentine (0.1 mL) was injected into the left thigh muscle of female Kunming mice (18–22 g). Animal studies were performed according to the principles of laboratory animal care and the guidelines of the Ethics Committee of Beijing Normal University (permit no. CLS-EAW-2018-001, June 2018). Approximately 5–7 days later, the mice were injected with 7.4 × 10^5^ Bq of the ^99m^Tc-labeled complexes ([^99m^Tc]Tc-CN4NF or [^99m^Tc]Tc-CN5NF) via the tail vein. The mice were sacrificed at 4 h postinjection. The organs of interest (the infected section, normal muscle in the right thigh, blood, heart, liver, lung, kidney, spleen, stomach, bone, and small intestine) were collected, weighed, and quantified for radioactivity. The results are expressed as the percentage of injected dose per gram of tissue (% ID/g). After the mice were sacrificed, the tissues containing thyroid around the trachea in the neck were taken and quantified for radioactivity. The results are expressed as the percentage of injected dose (%ID).

### 2.9. SPECT Imaging

A micro SPECT/CT scanner (Trifoil) with HiSPECT software and VivoQuant 2.5 software (Invicro, Boston, MA, USA) was used for SPECT/CT imaging studies. [^99m^Tc]Tc-CN5NF (0.1 mL, 18.5 MBq) was injected intravenously through the tail vein into a Kunming mouse bearing infection in the left thigh and inflammation in the right thigh. After anesthetization with 1.5% isoflurane in air, the mouse was fixed at the center of the SPECT scanner. SPECT image was obtained 120 min after [^99m^Tc]Tc-CN5NF injection.

### 2.10. Statistical and Data Analysis

All statistical analysis was performed with Microsoft Office Excel 2016. Quantitative data were presented as the mean ± standard deviation. Data were analyzed using an unpaired two-tailed Student’s *t* test. *p* < 0.05 represented statistically significant.

Information of the SPECT imaging reconstruction technique was described as follows: The OSEM reconstruction technique (the number of iterations was 2 and the number of subsets was 8) was used for SPECT. The SPECT image was opened with vivoquant 2.5 software and its maximum and minimum values were adjusted to a suitable value to make the images more readable (in vivo image: maximum value was 50,000, minimum value was 100) and any other processing methodologies were not implemented.

## 3. Results

### 3.1. Synthesis

The reactions schemes of the norfloxacin isonitriles (CN4NF and CN5NF) are depicted in Scheme 1. These compounds were prepared by reacting norfloxacin with different isonitrile-containing active esters (compounds **7** and **8**) under alkaline conditions. The final products were identified by ^1^H-NMR and ESI-MS (Related spectra for structural characterization were presented in Supplementary Materials Figures S1, S2, S3 and S4, respectively), and the results showed that the target ligands were successfully synthesized.

### 3.2. Radiolabeling

**Figure 2:**
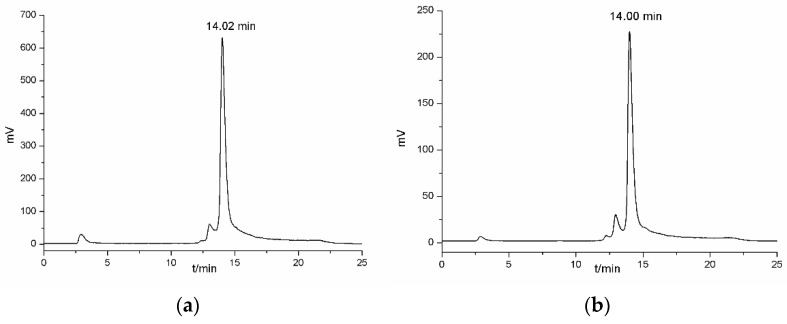
(**a**) HPLC profiles of [^99m^Tc]Tc-CN4NF and (**b**) [^99m^Tc]Tc-CN5NF.

[^99m^Tc]Tc-CN4NF and [^99m^Tc]Tc-CN5NF were easily prepared in high yields through a simple reaction without further purification. The radiochemical purities of the complexes were determined by TLC and HPLC. The TLC results of the complexes were as follows. In ACD solution, [^99m^Tc]TcO_4_^−^ and [^99m^Tc]TcO_2_.nH_2_O were moved with the solvent front, while [^99m^Tc]Tc-CN4NF and [^99m^Tc]Tc-CN5NF remained at the origin. The HPLC results of the complexes are shown in Figure 2. The retention times of [^99m^Tc]Tc-CN4NF and [^99m^Tc]Tc-CN5NF were 14.02 min and 14.00 min, respectively. Under the same conditions, the retention time of [^99m^Tc]TcO_4_^−^ was 3.93 min [30]. The HPLC analysis results showed that the RCPs of the radiotracers were more than 90%.

### 3.3. Physicochemical Properties Evaluation

#### 3.3.1. Stability Tests

Both [^99m^Tc]Tc-CN4NF and [^99m^Tc]Tc-CN5NF were stable in saline for 6 h and in mouse serum for 6 h, as examined by TLC. The RCPs of the radiotracers were more than 90% under those conditions. Almost no decomposition of the radiolabeled complexes was found, suggesting their great stabilities in vitro.

#### 3.3.2. Partition Coefficients

The partition coefficient (log *p*) values of [^99m^Tc]Tc-CN4NF and [^99m^Tc]Tc-CN5NF were −2.43 ± 0.10 and −1.75 ± 0.03, respectively. The results demonstrated that both tracers were hydrophilic. Moreover, [^99m^Tc]Tc-CN4NF was more hydrophilic than [^99m^Tc]Tc-CN5NF.

### 3.4. In Vitro Binding of [^99m^Tc]Tc-CN4NF and [^99m^Tc]Tc-CN5NF with Bacteria

**Figure 3:**
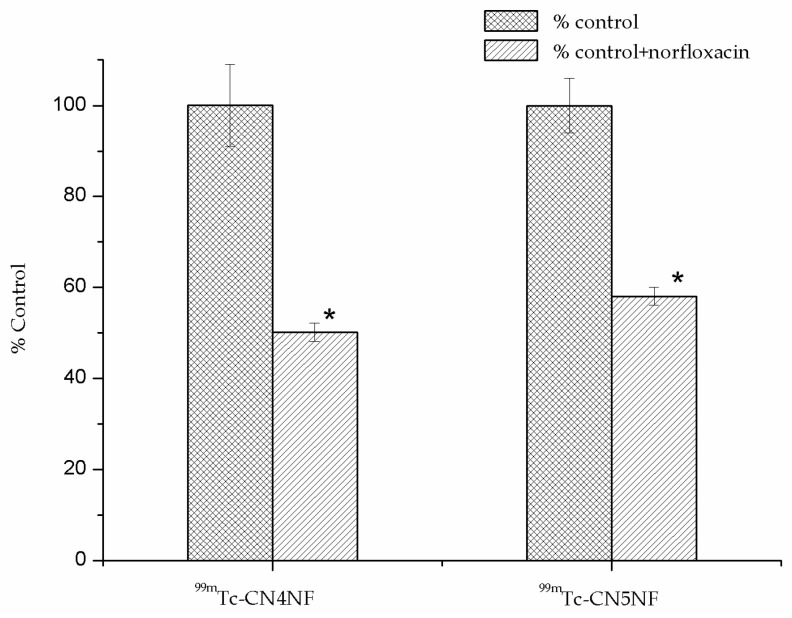
In vitro binding of [^99m^Tc]Tc-CN4NF and [^99m^Tc]Tc-CN5NF. *n* = 6, * *p* < 0.05.

The results of the in vitro bacteria (*S. aureus*) binding study of [^99m^Tc]Tc-CN4NF and [^99m^Tc]Tc-CN5NF are shown in Figure 3. Excess norfloxacin was added for competition. The binding values of the radiotracers to bacteria were set to 100% as control groups and the data of binding of [^99m^Tc]Tc-CN4NF and [^99m^Tc]Tc-CN5NF to the bacteria competing with norfloxacin are shown as the binding value/control group ratio. The results indicated that the binding of the complexes to the bacteria was significantly reduced. The binding values of [^99m^Tc]Tc-CN4NF and [^99m^Tc]Tc-CN5NF were reduced by 49.91% and 41.98%, respectively. This result suggested that both of these compounds specifically bound to bacteria.

### 3.5. Biodistribution Assessment

**Figure 4:**
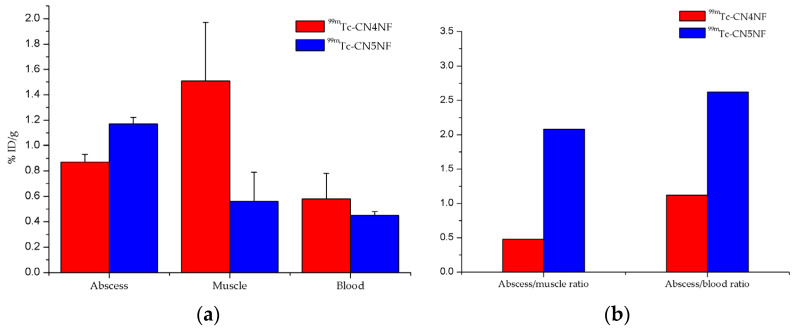
Comparison of the biodistribution in mice of [^99m^Tc]Tc-CN4NF and [^99m^Tc]Tc-CN5NF. (**a**) Infection uptake of the complexes at 4 h post-injection; (**b**) target/non-target ratio of the complexes at 4 h post-injection.

The biological distribution results in mice bearing bacterial infections and turpentine-induced abscesses are shown in Table 1. Comparisons of the two technetium-99m complexes are shown in Figure 4.

As seen in Table 1, in the bacteria-infected mice, the infection uptake values of [^99m^Tc]Tc-CN4NF and [^99m^Tc]Tc-CN5NF were 0.87 ± 0.06% ID/g and 1.17 ± 0.05% ID/g at 4 h post-injection, respectively, suggesting that these compounds were taken up by the abscesses and the radioactivity was retained well in the infection foci. Fast blood activity clearance was observed between 0.5 h and 4 h post-injection.

In addition, the abscess uptake values of [^99m^Tc]Tc-CN4NF and [^99m^Tc]Tc-CN5NF in mice with turpentine-induced abscesses was lower than that in bacteria-infected mice. Their abscess uptake was 0.53 ± 0.03% ID/g and 0.69 ± 0.37% ID/g at 4 h post-injection, respectively. The abscess/muscle and abscess/blood ratios at 4 h post-injection were also lower than those in the bacteria-infected mice.

As demonstrated in Figure 4, the uptake values of [^99m^Tc]Tc-CN5NF in muscle (0.56 ± 0.23) and blood (0.45 ± 0.03) at 4 h post-injection were lower than the uptake values of [^99m^Tc]Tc-CN4NF (muscle 1.51 ± 0.46 and blood 0.58 ± 0.20). Since [^99m^Tc]Tc-CN5NF exhibited significantly higher abscess/muscle and abscess/blood ratios, the infection uptake of [^99m^Tc]Tc-CN5NF was higher than that of [^99m^Tc]Tc-CN4NF.

For other organs, the low values of radioactivity uptake in the stomach and thyroid indicated that the complexes had good stability in vivo.

### 3.6. SPECT/CT Imaging Studies

**Figure 5:**
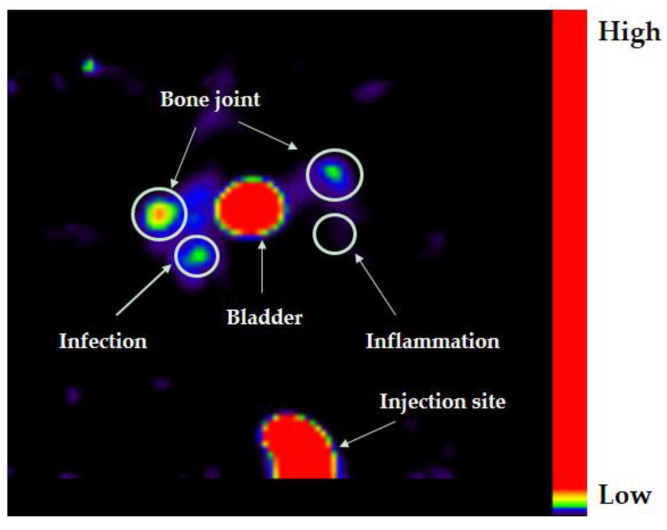
Half-body SPECT image of [^99m^Tc]Tc-CN5NF in female Kunming mice bearing infection and inflammation at 2 h post-injection (visual imaging).

According to the biodistribution results, [^99m^Tc]Tc-CN5NF was selected as a promising candidate for further SPECT/CT imaging, and the images are shown in Figure 5. SPECT imaging of mice bearing two different inflammation models indicated that the radioactivity associated with [^99m^Tc]Tc-CN5NF was localized at the site of infection. The ROI value showed that the signal at the bacterial infection site was 2-fold higher than that at sterile inflammation site, which was in accordance with the biodistribution results in mice. The imaging results suggested that [^99m^Tc]Tc-CN5NF exhibited potential as an infection imaging agent.

## 4. Discussion

In recent decades, the development of SPECT/CT hardware has greatly improved. Among them, the successful manufacture of cadmium–zinc–telluride (CZT) detectors promotes the sensitivity and resolution of SPECT/CT to offer accurate absolute radiotracer uptake quantification compared to PET/CT [31]. Technetium-99m, as a generator-produced SPECT radionuclide, is a very good choice for radiopharmaceuticals due to its ideal nuclide properties, availability, and affordability. Thus, the development of novel ^99m^Tc-labeled norfloxacin derivatives as bacterial infection imaging agents has great significance. Generally, the majority of ^99m^Tc radiopharmaceuticals are designed through a bifunctional approach [32]. There are three sections (a pharmacophore, a chelating group, and a linking group) in each ligand, which are used to prepare the ^99m^Tc-labeled bifunctional radiopharmaceuticals. The pharmacophore qualifies the target, while the chelating group can form a complex with ^99m^Tc. The pharmacophore and the chelating group are connected together to form the ^99m^Tc complex by a linker, which may have an impact on the pharmacokinetic properties of the radiotracers. Therefore, when both the pharmacophore and chelating group are determined and unchanged, it is considered that the linker can be modified to fix the pharmacokinetic properties to produce an ideal radiopharmaceutical. Lipid solubility has a significant impact on the biodistribution of ^99m^Tc radiotracers. The lipophilicity of the complexes can adjust the penetration of the radiotracers into the lipophilic membrane of bacterial cells. The uptake of the complex at the infection sites can be strengthened by increasing the lipid solubility. Isonitrile (CN-R), as a bifunctional agent, is able to coordinate with the [^99m^Tc]Tc(I) core to prepare [^99m^Tc][Tc(CN-R)_6_]^+^ in high yield. For norfloxacin isonitrile derivatives, in the structure of norfloxacin, carbonyl and carboxyl groups are considered necessary pharmacophores. In order to retain the pharmacophore of norfloxacin, which specifically binds to bacteria, we chose the piperazinyl group of norfloxacin to react with different isonitrile-containing active esters to obtain different isonitrile derivatives (CN4NF and CN5NF). The difference between these designed compounds is that CN5NF possesses one additional methylene moiety.

The preparation of [^99m^Tc]Tc-CN4NF and [^99m^Tc]Tc-CN5NF was easy and convenient. The RCPs of the products were over 90%, and there was no need for further purification. The structures of the complexes are similar to that of [^99m^Tc]Tc-MIBI [33], having six CN4NF or CN5NF ligands around technetium-99m (+1) to form a regular octahedron structure. The structure was stable; thus, nearly no decomposition of [^99m^Tc]Tc-CN4NF or [^99m^Tc]Tc-CN5NF was found after remaining at room temperature in saline and in 37 °C and in mouse serum for one half-life of ^99m^Tc (6 h).

On the basis of the partition coefficient results, [^99m^Tc]Tc-CN4NF (log *p* = −2.43 ± 0.10) and [^99m^Tc]Tc-CN5NF (log *p* = −1.75 ± 0.03) were hydrophilic complexes. [^99m^Tc]Tc-CN4NF showed greater hydrophilicity than [^99m^Tc]Tc-CN5NF because CN4NF has a shorter carbon chain length than CN5NF. The linker moiety, such as hydrocarbon chains (CH_2_)_n_, can change the lipophilicity of the complex.

In an in vitro bacterial binding assay, the competition results showed that bacterial binding decreased by more than 40%, which showed that the specific binding of the two complexes to bacteria was inhibited by norfloxacin.

The biodistribution study indicated that [^99m^Tc]Tc-CN4NF accumulated in infected foci (0.87% ID/g) at 4 h post-injection, and the abscess/blood and abscess/muscle ratios were 1.50 and 0.58, respectively. However, abscess uptake in mice with turpentine-induced abscesses (0.53% ID/g) at 4 h post-injection was lower. For [^99m^Tc]Tc-CN5NF, the uptake in infected sections was 1.17% ID/g at 4 h post-injection, which was higher than that in mice with sterile inflammation (0.69% ID/g). The abscess/blood and abscess/muscle ratios were 2.62 and 2.08, respectively. Comparing the results of the biodistribution of infected and inflammatory mice, we found a significant difference in the uptake of the two radiolabeled complexes between the infected and inflammatory foci.

Compared to [^99m^Tc]Tc-CN4NF, [^99m^Tc]Tc-CN5NF had higher infection accumulation at 0.5 h, 2 h, and 4 h post-injection. Perhaps [^99m^Tc]Tc-CN5NF is more lipophilic, which may enhance transudation of the radiotracer at the infected site. For the muscle at 4 h post-injection, the uptake of [^99m^Tc]Tc-CN4NF (1.51% ID/g) was nearly three times as much as that of [^99m^Tc]Tc-CN5NF (0.51% ID/g). The uptake in blood remained basically the same, and we found fast clearance from the blood between 0.5 h and 4 h post-injection (2.17% ID/g reduced to 0.58% ID/g and 1.87% ID/g reduced to 0.45% ID/g for [^99m^Tc]Tc-CN4NF and [^99m^Tc]Tc-CN5NF, respectively). Therefore, the target/non-target ratio of [^99m^Tc]Tc-CN5NF was much higher than that of [^99m^Tc]Tc-CN4NF. At 4 h post-injection, the abscess/blood and abscess/muscle ratios were 2.62 and 2.08, respectively, while the corresponding [^99m^Tc]Tc-CN4NF data were 1.50 and 0.58, respectively. For other non-target organs, the radioactive accumulation in the liver (14.18% ID/g) of [^99m^Tc]Tc-CN4NF was lower than that of [^99m^Tc]Tc-CN5NF (16.23% ID/g). According to the partition coefficient data of these two compounds, [^99m^Tc]Tc-CN4NF was more hydrophilic, which possibly caused lower uptake in the liver. The radioactivity uptake values of the complexes into the stomach and thyroid were low, which demonstrated that they both had good stability in vivo. For comparison, [^99m^Tc]Tc-CN5NF had lower uptake in the lung, kidneys, spleen, and bone. In this case, [^99m^Tc]Tc-CN5NF may obtain higher-quality abdominal images than [^99m^Tc]Tc-CN4NF.

NFXDTC is a dithiocarbamate norfloxacin derivative that can conjugate with the [^99m^Tc][TcN]^2+^ core to obtain a stable complex in the form of [^99m^Tc]TcN(L)_2_ (L = bidentate ligand). In the biodistribution study, the infection uptake of [^99m^Tc]TcN-NFXDTC (3.43% ID/g) was higher than that of [^99m^Tc]Tc-CN5NF (1.17% ID/g) at 4 h post-injection [26]. Probably, different strains of *S. aureus* caused different infected mouse models, which resulted in comparative infection accumulation. In addition, the uptake of [^99m^Tc]Tc-CN5NF in muscle (0.51% ID/g) and blood (0.45% ID/g) was lower than that of [^99m^Tc]TcN-NFXDTC (0.99% ID/g and 0.84% ID/g). For non-target organs, the radioactive accumulation of [^99m^Tc]TcN-NFXDTC in the lung (22.87% ID/g) and the liver (28.52% ID/g) were considerable, while the lung (0.93% ID/g) and liver (16.23% ID/g) uptake of [^99m^Tc]Tc-CN5NF were much lower. Thus, during SPECT imaging, [^99m^Tc]Tc-CN5NF would have less interference because of the lower uptake in the lung, which may lead to better images.

Because of its admirable biodistribution performance, [^99m^Tc]Tc-CN5NF was selected for a further SPECT imaging study. As predicted, [^99m^Tc]Tc-CN5NF was able to distinguish bacterial infection from sterile inflammation. The results suggested that [^99m^Tc]Tc-CN5NF has the potential to be used in molecular imaging to diagnose infection and monitor treatment efficiency.

## 5. Conclusions

In this work, norfloxacin isonitrile derivatives (CN4NF and CN5NF) were successfully prepared and labeled with a [^99m^Tc][Tc(I)]^+^ core to obtain [^99m^Tc]Tc-CN4NF and [^99m^Tc]Tc-CN5NF in high yields. The two radiolabeled complexes were hydrophilic and showed good stability in vitro and specificity to bacteria. Among them, the biodistribution results in mice suggested that [^99m^Tc]Tc-CN5NF exhibited a higher infection uptake and a higher target/non-target ratio. The SPECT imaging study demonstrated that [^99m^Tc]Tc-CN5NF can differentiate between bacterial infection and sterile inflammation. [^99m^Tc]Tc-CN5NF shows potential as a good bacterial infection imaging tracer, justifying further investigations.

## Figures and Tables

**Table 1:** Biodistribution of [^99m^Tc]Tc-CN4NF and [^99m^Tc]Tc-CN5NF in mice (% ID/g ± SD, *n* = 4).

	[^99m^Tc]Tc-CN4NF				[^99m^Tc]Tc-CN5NF			
	Mice with Bacterial Infection			Mice with Turpentine-Induced Abscess	Mice with Bacterial Infection			Mice with Turpentine-Induced Abscess
Tissue	0.5 h	2 h	4 h	4 h	0.5 h	2 h	4 h	4 h
Heart	1.90 ± 0.23	0.74 ± 0.15	0.75 ± 0.12	1.05 ± 0.06	1.77 ± 0.35	0.98 ± 0.16	0.87 ± 0.10	1.12 ± 0.44
Liver	12.55 ± 1.08	14.02 ± 1.04	14.18 ± 0.39	14.21 ± 1.26	14.13 ± 1.43	15.51 ± 0.88	16.23 ± 1.17	16.63 ± 0.53
Lung	4.99 ± 0.28	1.76 ± 0.15	1.46 ± 0.03	1.61 ± 0.37	3.59 ± 0.60	1.75 ± 0.49	0.93 ± 0.08	1.26 ± 0.70
Kidneys	6.53 ± 1.15	5.12 ± 0.61	6.00 ± 0.24	5.42 ± 0.71	5.71 ± 0.80	4.28 ± 0.24	3.87 ± 0.07	2.97 ± 1.03
Spleen	5.97 ± 1.12	7.11 ± 1.44	5.11 ± 0.82	6.79 ± 2.05	4.89 ± 0.83	3.80 ± 0.66	2.98 ± 0.75	3.46 ± 0.83
Stomach	1.59 ± 0.14	1.60 ± 0.26	1.37 ± 0.51	1.01 ± 0.17	5.02 ± 0.82	4.53 ± 0.75	4.32 ± 0.88	1.15 ± 0.24
Bone	12.08 ± 1.43	12.31 ± 1.05	14.13 ± 1.10	12.20 ± 2.23	8.18 ± 0.91	9.33 ± 1.45	8.25 ± 0.20	8.91 ± 1.10
Muscle	2.71 ± 0.37	2.12 ± 0.65	1.51 ± 0.46	1.10 ± 0.30	1.43 ± 0.16	0.98 ± 0.14	0.56 ± 0.23	0.73 ± 0.08
Intestine	1.41 ± 0.11	0.80 ± 0.16	0.73 ± 0.18	0.81 ± 0.16	1.52 ± 0.18	0.64 ± 0.22	0.68 ± 0.22	0.44 ± 0.16
Abscess	1.89 ± 0.73	1.15 ± 0.27	0.87 ± 0.06	0.53 ± 0.03	1.94 ± 0.19	1.47 ± 0.08	1.17 ± 0.05	0.69 ± 0.37
Blood	2.17 ± 0.38	0.74 ± 0.11	0.58 ± 0.20	0.47 ± 0.08	1.87 ± 0.23	0.74 ± 0.07	0.45 ± 0.03	0.45 ± 0.10
Thyroid (%ID)	0.10 ± 0.02	0.10 ± 0.03	0.08 ± 0.02	0.07 ± 0.01	0.05 ± 0.01	0.05 ± 0.01	0.03 ± 0.01	0.03 ± 0.00
T/N ^1^	0.70	0.54	0.58	0.48	1.36	1.50	2.08	0.95
T/B ^2^	0.87	1.55	1.50	1.12	1.04	1.98	2.62	1.55

^1^ T/N = abscess/muscle ratio. ^2^ T/B = abscess/blood ratio.

## Data Availability

Data is contained within the article or Supplementary Material.

## Supplementary Materials

The following are available online at https://www.mdpi.com/article/10.3390/pharmaceutics13040518/s1, synthesis of Compound **9** and Compound **10**, Figure S1: ^1^H NMR spectrum of Compound **9**, Figure S2: ^1^H NMR spectrum of Compound **10**, Figure S3: HR-MS spectrum of Compound **9**, Figure S4: HR-MS spectrum of Compound **10**.
