# Supplementary material for: Synthesis and Evaluation of Novel Norfloxacin Isonitrile 99mTc Complexes as Potential Bacterial Infection Imaging Agents
Source: Pharmaceutics. 2021 Apr 9;13(4):518. doi: 10.3390/pharmaceutics13040518 (PMC8069222; doi:10.3390/pharmaceutics13040518)
Supplement: Supplementary file 1 [file pharmaceutics-13-00518-s001.pdf]

pharmaceutics

Supplementary Materials: Synthesis and evaluation of novel norfloxacin isonitrile ^{99m}Tc complexes as potential bacterial infection imaging agents

Si'an Fang, Yuhao Jiang, Di Xiao, Xuran Zhang, Qianqian Gan, Qing Rua and Junbo Zhang

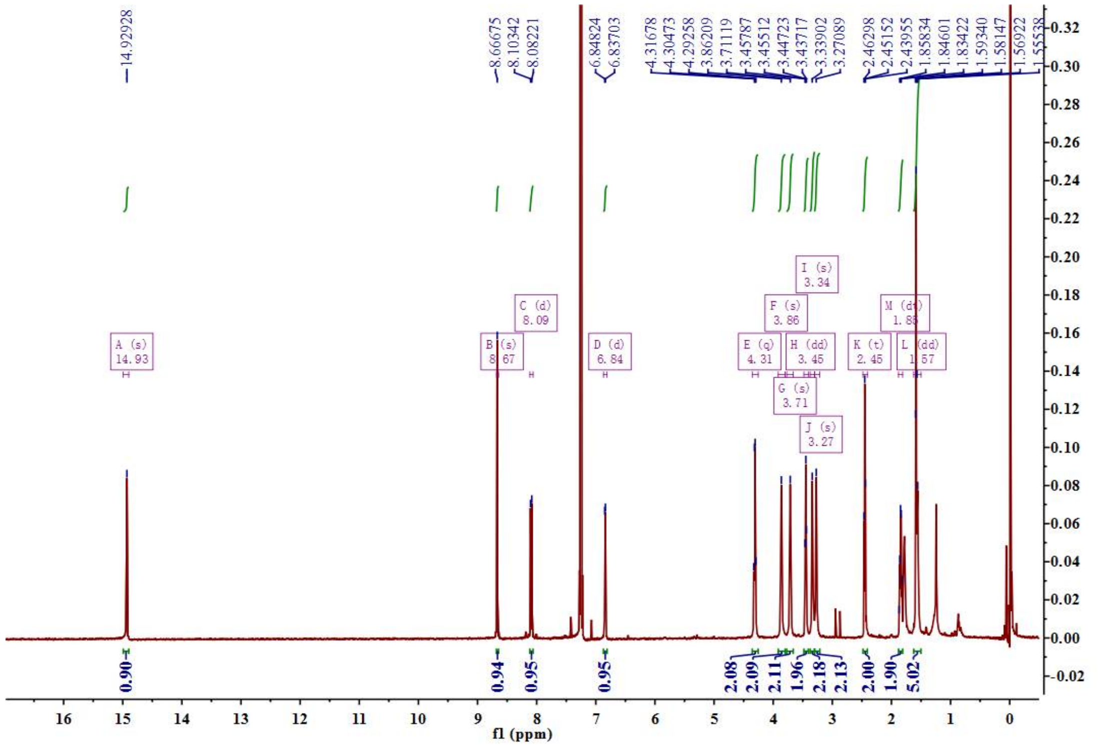

Figure S1. ^{1}H NMR spectrum of Compound **9** (CN4NF).

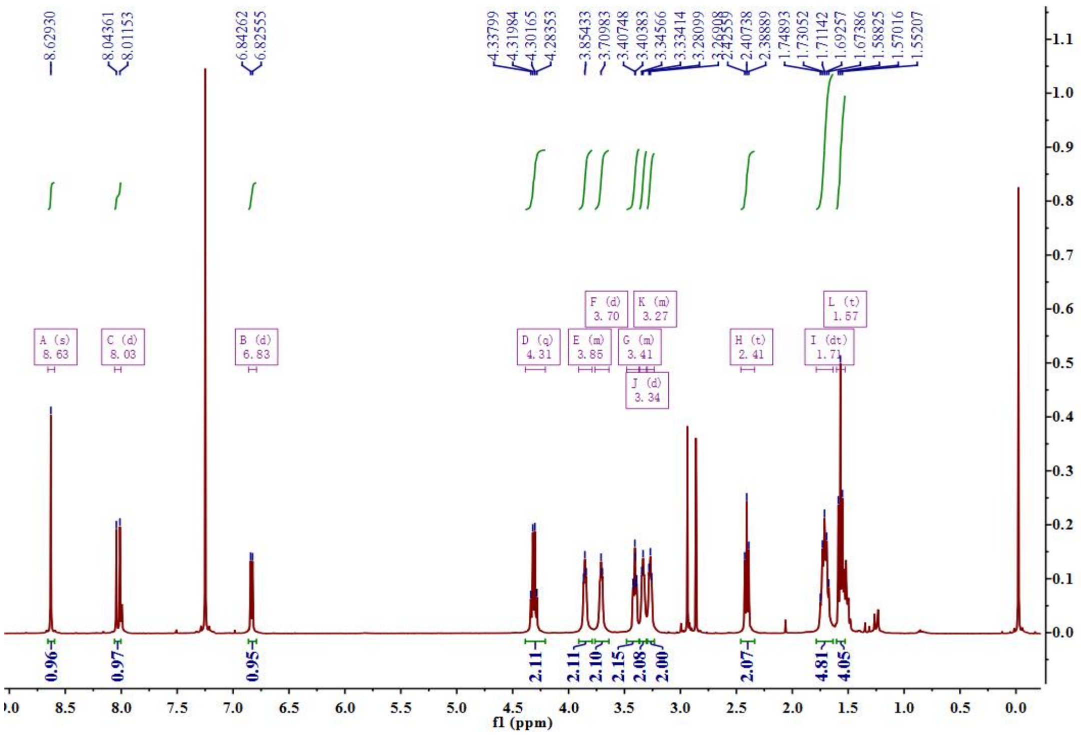

Figure S2. ^{1}H NMR spectrum of Compound **10** (CN5NF).

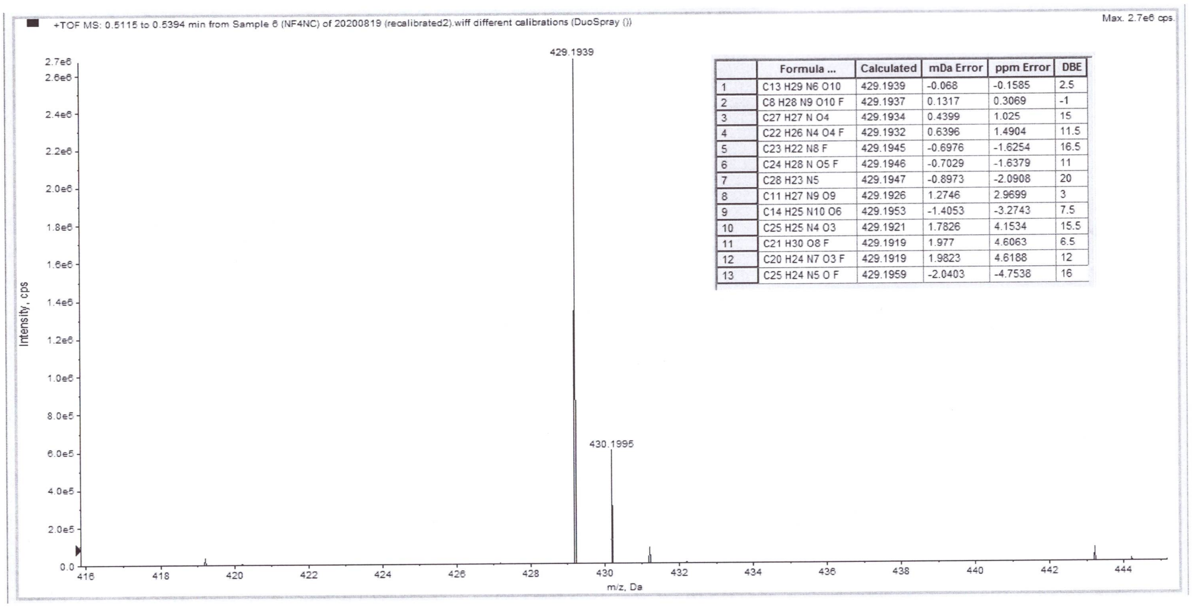

	Formula ...	Calculated	mDa Error	ppm Error	DBE
1	C13 H29 N6 O10	429.1939	-0.068	-0.1585	2.5
2	C8 H28 N9 O10 F	429.1937	0.1317	0.3069	-1
3	C27 H27 N O4	429.1934	0.4399	1.025	15
4	C22 H26 N4 O4 F	429.1932	0.6396	1.4904	11.5
5	C23 H22 N8 F	429.1945	-0.6976	-1.6254	16.5
6	C24 H28 N O5 F	429.1946	-0.7029	-1.6379	11
7	C28 H23 N5	429.1947	-0.8973	-2.0908	20
8	C11 H27 N9 O9	429.1926	1.2746	2.9699	3
9	C14 H25 N10 O6	429.1953	-1.4053	-3.2743	7.5
10	C25 H25 N4 O3	429.1921	1.7826	4.1534	15.5
11	C21 H30 O8 F	429.1919	1.977	4.6063	6.5
12	C20 H24 N7 O3 F	429.1919	1.9823	4.6188	12
13	C25 H24 N5 O F	429.1959	-2.0403	-4.7538	16

Figure S3. HR-MS spectrum of Compound **9** (CN4NF).

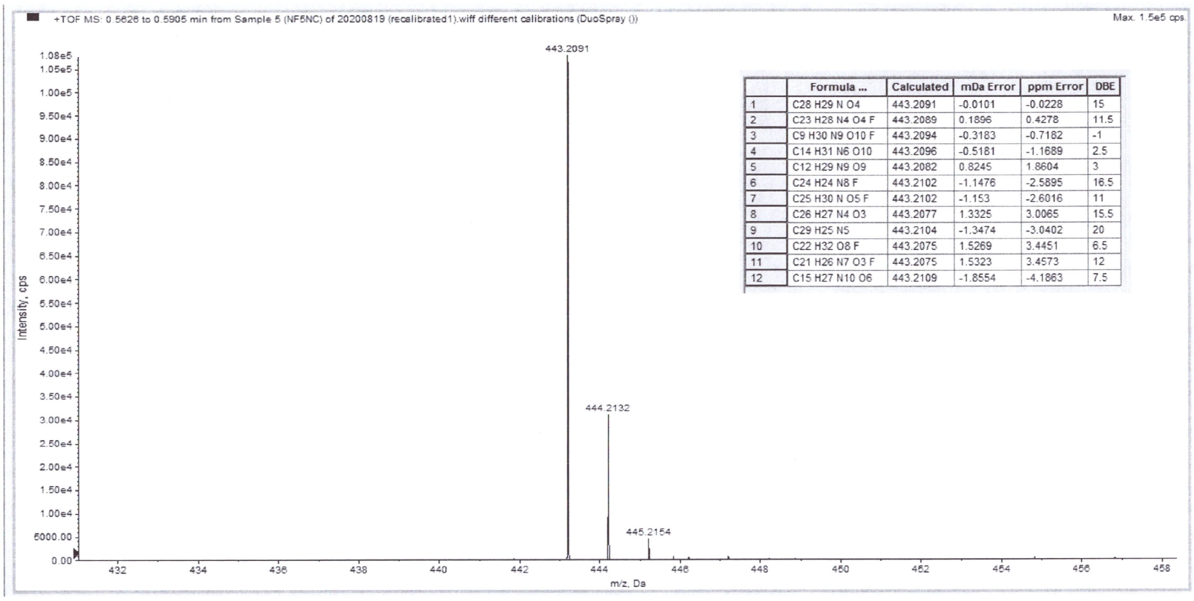

	Formula ...	Calculated	mDa Error	ppm Error	DBE
1	C28 H29 N O4	443.2091	-0.0101	-0.0228	15
2	C23 H28 N4 O4 F	443.2089	0.1896	0.4278	11.5
3	C9 H30 N9 O10 F	443.2094	-0.3183	-0.7182	-1
4	C14 H31 N6 O10	443.2096	-0.5181	-1.1689	2.5
5	C12 H29 N9 O9	443.2082	0.8245	1.8604	3
6	C24 H24 N8 F	443.2102	-1.1476	-2.5895	16.5
7	C25 H30 N O5 F	443.2102	-1.153	-2.6016	11
8	C26 H27 N4 O3	443.2077	1.3325	3.0065	15.5
9	C29 H25 N5	443.2104	-1.3474	-3.0402	20
10	C22 H32 O8 F	443.2075	1.5269	3.4451	6.5
11	C21 H26 N7 O3 F	443.2075	1.5323	3.4573	12
12	C15 H27 N10 O6	443.2109	-1.8554	-4.1863	7.5

Figure S4. HR-MS spectrum of Compound **10** (CN5NF).
